# Effect of manual aspiration thrombectomy using large-bore aspiration catheter for acute basilar artery occlusion: comparison with a stent retriever system

**DOI:** 10.1186/s12883-020-02013-7

**Published:** 2020-11-30

**Authors:** Jin Wook Choi, Miran Han, Jung Hyun Park, Woo Sang Jung

**Affiliations:** Department of Radiology, Ajou University School of Medicine, Ajou University Medical Center, Suwon, South Korea

**Keywords:** Large-bore aspiration catheter, Manual aspiration thrombectomy, Acute basilar artery occlusion, First-pass effect, Puncture-to-recanalization time

## Abstract

**Background:**

A large-bore aspiration catheter can be employed for recanalization of acute basilar artery occlusion. Here we compare the results of mechanical thrombectomy using a stent retriever (SR) and manual aspiration thrombectomy (MAT) using a large-bore aspiration catheter system as a first-line recanalization method in acute basilar artery occlusion (BAO).

**Methods:**

The records of 50 patients with acute BAO who underwent mechanical thrombectomy were retrospectively reviewed. Patients were assigned to one of two groups based on the first-line recanalization method. The treatment and clinical outcomes were compared.

**Results:**

Sixteen (32%) patients were treated with MAT with a large-bore aspiration catheter and 34 (68%) with a SR as the first-line treatment method. The MAT group had a shorter procedure time (28 vs. 65 min; *p* = 0.001), higher rate of first-pass recanalization (68.8% vs. 38.2%, *p* = 0.044), and lower median number of passes (1 vs 2; *p* = 0.008) when compared with the SR group. There was no significant difference in the incidence of any hemorrhagic complication (6.3% vs. 8.8%; *p* = 0.754) between the groups. However, there were four cases of procedure-related subarachnoid hemorrhage (SAH) in the SR group and one death occurred due to massive hemorrhage.

**Conclusions:**

Selection of MAT using a large-bore aspiration catheter for acute BAO may be a safe and effective first-line treatment method with higher first-pass recanalization rate and shorter procedure time than SR.

## Background

Acute basilar artery occlusion (BAO) is a rare condition, accounting for about 1% of all strokes [1]. Despite advances in acute ischemic stroke treatment recently, the prognosis remains poor with a high mortality rate in patients with BAO, especially in the absence of early reperfusion [2].

Mechanical thrombectomy (MT) techniques have evolved rapidly in an effort to improve the rate of successful recanalization for acute ischemic stroke due to large vessel occlusion (LVO) [3–7]. The main methods of MT for LVO include stent retriever (SR) thrombectomy or manual aspiration thrombectomy (MAT) with a large-bore distal access catheter. There have been several recent studies comparing the efficacy and safety of the catheter aspiration technique and the standard stent retriever technique as a fist-line endovascular treatment in patients with anterior circulation LVO [8, 9]. However, there is a paucity of data on the role of MAT using a large-bore catheter compared with SR thrombectomy for patients with BAO.

In this study, we aimed to compare the efficacy and safety of MAT using a large-bore catheter and that of SR in patients with BAO.

## Methods

### Patients

This study was a single-center, retrospective analysis of patients who were treated with SR or MAT as a first-line method for acute BAO from March 2016 to December 2019. The institutional review board approved this study (AJIRB-MED-MDB-20-084) and waived the requirement for written informed consent based on the retrospective study design.

All patients with a clinical diagnosis of acute ischemic stroke (AIS) due to basilar artery occlusion who underwent initiation of endovascular stroke treatment with complete angiographic evaluation were included. There was no predetermined protocol for the treatment of BAO with endovascular procedure at our hospital during the study period. The decision to initiate endovascular treatment in any given patient with BAO on presentation was left to the attending neurointerventional specialist’s discretion in agreement with the neurologist according to neurological guidelines.

All patients with BAO who arrived at our hospital underwent an initial imaging protocol including non-enhanced CT, CT angiography or MRI. During the study period, 52 patients who underwent endovascular treatment for acute BAO were initially enrolled. Among them, two patients with occlusion due to dissection were excluded. The remaining 50 patients were included in final analysis.

### Endovascular procedure

All procedures were performed by experienced neurointerventional radiologists and neurologists using a biplanar Allura Xper FD scanner (Philips Healthcare). Angiography was performed via the femoral approach using a 5-F angiographic catheter. An initial angiographic series from both vertebral arteries was obtained to confirm the location of the occlusion and to evaluate the best access to the occlusion site. Then, a 6-Fr shuttle catheter (Cook, Bloomington, IN, USA) was placed in the distal portion of the V2 segment of the dominant vertebral artery (VA) via a co-axial delivery method using an angiographic catheter and guidewire.

**Fig. 1:**
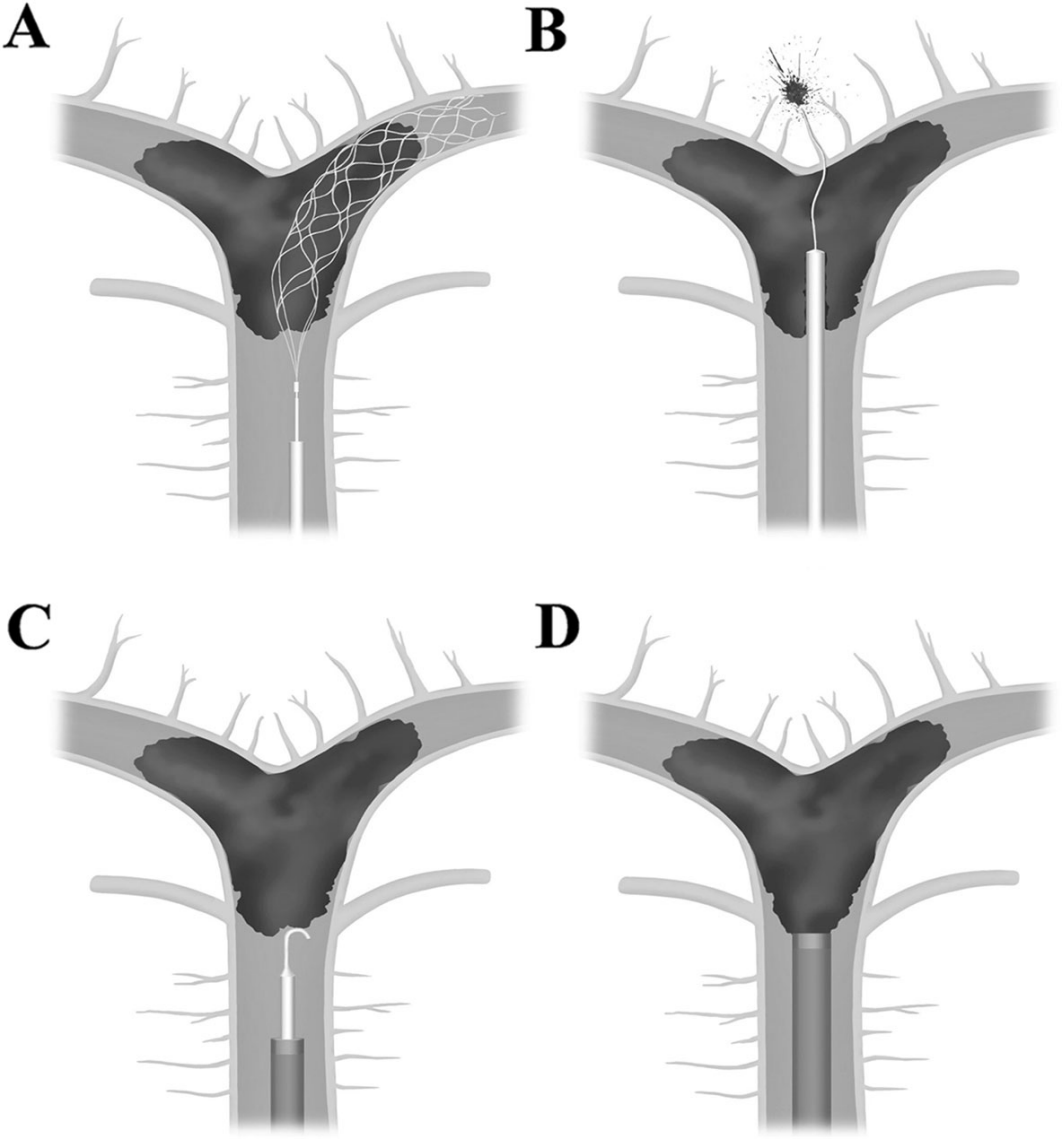
Schematic drawing of mechanical thrombectomy using SR and MAT. **a** The SR stent should be deployed where it can completely cover the occluded segment. For complete coverage, the guidewire must be blindly passed through the occluded segment. **b** Blind wire passage for mechanical thrombectomy using SR has a risk of vascular perforation. **c**, **d** When MAT was performed, the guide wire and large-bore aspiration catheter are located at the proximal end of the clot, which minimizes the risk of vascular injury. (This illustration was made by ourselves)

For SR thrombectomy, a microcatheter (Prowler Select Plus; Cordis Neurovascular, Miami Lages, FL, USA) with a 0.014 in. microguidewire (Traxcess; Microvention, Tustin, CA, USA) was passed through the occlusion site and placed distally. A retrievable stent (Solitaire; Covidine, Irvine, CA or Trevo; Stryker, Kalamazoo, MI) was introduced through the microcatheter and after full deployment was held in position for 3–5 min to engage the clot (Fig. 1a). All the stents we used were 4/20 mm in size and length. Subsequently, the microcatheter and stent were pulled back, with application of negative suction pressure through the Shuttle catheter, using a 50-mL syringe to minimize distal embolization.

For large bore suction catheter thrombectomy, a Penumbra 5MAX/ ACE 68 catheter (Penumbra, Alameda, CA) or Sofia 6F distal access catheter (MicroVention, Tustin, CA) with a microcatheter and a microwire was advanced coaxially to the proximal end of the thrombus to wedge the catheter next to the clot (Fig. 1c, d). After withdrawal of the microcatheter and microwire, we connected a 50-mL syringe to the proximal end of the reperfusion catheter for manual aspiration. The catheter was withdrawn slowly, maintaining a vacuum between the tip and the clot with application of negative suction pressure through the Shuttle catheter, also using the 50-mL syringe. If the vacuum released during catheter withdrawal, we placed a large bore suction catheter on the proximal BA or distal VA and performed angiography via that catheter to explore whether recanalization had been successful. In cases of persistent occlusion or incomplete recanalization, the microcatheter and microwire were reintroduced and the procedure repeated until maximal recanalization was achieved.

After the procedure, all patients were treated in the intensive care unit. A routine non-enhanced CT was performed immediately after all procedures to rule out intracranial hemorrhage. Contrast-enhanced CT scans were performed 24 to 48 h after the procedure. Then, general supportive care was initiated, including intravenous antiplatelet or anticoagulation therapy, cardiac monitoring, control of blood pressure, fever, blood glucose and all other essential variables following stroke guidelines.

### Outcome measures

Clinical data including sex, age, medical risk factors (hypertension, diabetes mellitus, hyperlipidemia, smoking, atrial fibrillation, coronary artery disease, previous stroke history), NIHSS score on admission and at discharge, intravenous tissue plasminogen activator (IV tPA) administration, modified Rankin Scale (mRS) score 3 months after treatment, hemorrhage and mortality were obtained for evaluation of clinical outcomes. Procedure time was defined as time from puncture to final recanalization in successful recanalization cases and time of last angiographic series in unsuccessful recanalization cases. Recanalization status was assessed on the final angiogram and was classified according to the modified thrombolysis in cerebral infarction (mTICI) scale; successful recanalization was defined as modified TICI 2b or 3. First pass effect (FPE) was defined as achieving complete recanalization (mTICI 3) with a single thrombectomy device pass [10]. A favorable clinical outcome was defined as a three-month mRS score of 0–2. An intracerebral hemorrhage was classified according to the second European-Australasian Acute Stroke Study classification, and symptomatic ICH was defined as any CT-documented hemorrhage that was temporally related to deterioration in the patient’s clinical condition and a ≥ 4-point in NIHSS. All NIHSS and modified Rankin Score (mRS) grades were assessed by a consulting neurologist.

### Statistical analysis

All analyses were conducted using SPSS for Windows (version 25.0; IBM, Armonk, NY, USA). Descriptive statistics of normally distributed data are reported as means and standard deviations; non-normally distributed data are summarized as medians and interquartile ranges (IQR). Differences between groups were examined using Fisher’s exact test or the Mann-Whitney test. Multivariable logistic regression analysis was performed to identify variables independently associated with a favorable clinical outcome. Statistical significance was defined as *p* ≤ 0.05.

## Results

### Patients

Baseline characteristics are provided for the overall study population according to the first-line recanalization method (SR group vs. MAT group) in Table 1. Among 50 patients with acute BAO, 16 (32%) were treated with MAT with a large-bore aspiration catheter and 34 (68%) were treated with SR. Branching-site occlusions were more frequent in the MAT group (68.8% vs. 38.2%, *p* < 0.044). Other baseline characteristics were not significantly different between the two groups.

**Table 1:** Comparison of baseline characteristics of patients with basilar artery occlusion according to first-line thrombectomy method

	All patients, *n* = 50	First-line MAT, *n* = 16	First-line SR, *n* = 34	*P* value
Age, mean (range)	68.1 (18–89)	65.25 (51–84)	69.38 (18–89)	0.34
Sex (male)	26 (52.0)	10 (62.5)	16 (47.0)	0.308
Hypertension	28 (56.0)	6 (37.5)	22 (64.7)	0.071
Hypercholesterolemia	5 (10.0)	3 (18.7)	2 (5.8)	0.311
Diabetes	13 (26.0)	5 (31.2)	8 (23.5)	0.731
Smoking	4 (8.0)	1 (6)	3 (8.8)	0.754
Atrial fibrillation	14 (28.0)	7 (43.7)	7 (20.5)	0.089
Coronary artery disease	2 (4.0)	0 (0)	2 (5.8)	0.322
IV tPA	22 (44.0)	5 (31.2)	17 (50)	0.213
Admission NIHSS^a^	21 (13.25–25)	19.5 (13.5–22.25)	21.5 (13.25–26.75)	0.371
Branching-site occlusion	24 (48)	11 (68.8)	13 (38.2)	0.044

Values in parentheses represent the number of patients (%). ^a^Data are medians and the numbers in parentheses are IQRs. *MAT* Manual aspiration thrombectomy, *SR* Stent retriever, *IV tPA* Intravenous tissue-type plasminogen activator, *NIHSS* National Institutes of Health Stroke Scale

### Angiographic & clinical outcomes

**Fig. 2:**
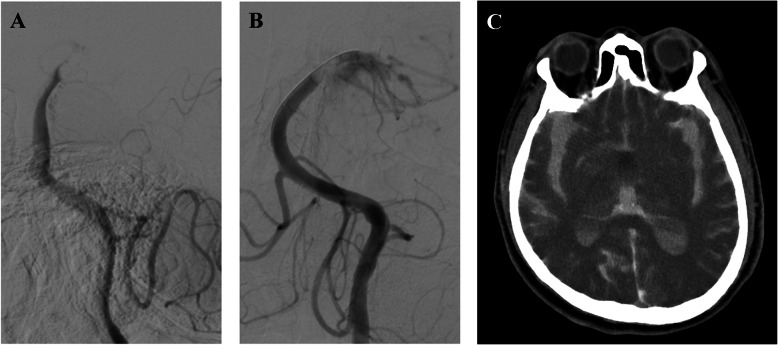
A 76-year-old man had a basilar artery occlusion. **a**, Angiography demonstrated complete occlusion of the distal basilar artery. **b**, After mechanical thrombectomy using a stent retriever, partial recanalization of the basilar artery was noted and extravascular contrast leakage was visible. **c**, Post-procedure CT showed subarachnoid and intraventricular hemorrhage

The angiographic and clinical outcomes are summarized in Table 2. Overall, successful recanalization was achieved in 78% (39/50) of patients and favorable clinical outcomes at 3 months in 42% (21/50) of patients. The MAT group had a shorter procedure time (28 vs. 65 min; *p* = 0.001), higher incidence of first pass effect (11/16, 68.8% vs. 13/34, 38.2%, *p* = 0.044), and lower total number of passes (median, 1 vs. 2; *p* = 0.008) when compared with the SR group. Successful recanalization and a favorable outcome at 3 months were more frequent in the MAT group but the differences did not reach statistical significance (14/16, 87.5% vs. 25/34, 73.5%; *p* = 0.466 and 9/16, 56.2% vs. 12/34, 35.3%; *p* = 0.161, respectively). There was no significant difference in the incidence of symptomatic ICH (1/16, 6.3% vs. 3/34, 8.8%; *p* = 0.754) between the groups. However, one patient in the SR group experienced a massive subarachnoid hemorrhage (SAH) with intraventricular extension because of basilar artery rupture during the procedure, which was evident on post-procedural CT (Fig. 2a, b, c). The good outcome rates defined as 3 month mRS score 0–2 were higher in the MAT group compared with the SR group irrespective of the location of the occlusion (Table 3).

**Table 2:** Comparison of treatment and clinical outcomes of patients with basilar artery occlusion according to first-line thrombectomy method

	All patients *n* = 50	First-line MAT *n* = 16	First-line SR *n* = 34	*P* value
Onset to puncture time (min)*^a^	137 (70–353)	125 (60–330)	140 (75–375)	0.34
Procedure time (min)^a^	55 (30–80)	28 (20–54)	65 (50–89)	0.001
Onset to recanalization time (min)^a^	197 (122–396)	168 (93–367)	202 (135–498)	0.383
First-pass effect	25 (50)	11 (68.8)	13 (38.2)	0.044
Adjuvant treatment				
Angioplasty	8 (16)	3 (18.8)	5 (14.7)	0.699
IA tirofiban	18 (36)	4 (25.0)	14 (41.2)	0.266
Number of passes^a^	1.5 (1–2)	1 (1–1.25)	2 (1–3)	0.008
mTICI 2b or 3	39 (78)	14 (87.5)	25 (73.5)	0.466
90d mRS^a^	4 (1.25–5)	2 (0–4.25)	4 (2–5)	0.043
90d mRS 0–2	21 (42)	9 (56.2)	12 (35.3)	0.161
Mortality	6 (12)	0 (0)	6 (17.6)	0.076
Symptomatic ICH	4 (8)	1 (6.3)	3 (8.8)	0.754
SAH	4 (8)	0 (0)	4 (11.7)	0.322

Values in parentheses represent the number of patients (%). ^a^Data are medians and the numbers in parentheses are IQRs. *IA* Intra-arterial, *ICH* Intracerebral hemorrhage, *mRS* Modified Rankin Scale, *mTICI* Modified thrombolysis in cerebral infarction

**Table 3:** Comparison of good outcome rate according to the occlusion site and first line treatment methods

	Proximal occlusion	Middle occlusion	Distal occlusion
First line MAT	2/4 (50)	1/1 (100)	6/11 (54.5)
First line SR	1/8 (12.5)	6/13 (46.1)	5/13 (38.5)

Values in parentheses represent the percentage of patients

Multivariate logistic regression analysis showed that the procedure time (OR 0.972, 95% CI 0.947 to 0.997; *p* = 0.029) and initial NIHSS score (OR 0.893, 95% CI 0.806 to 0.990; *p* = 0.031) were independent predictors of good clinical outcome after adjusting for relevant covariates (Table 4).

**Table 4:** Multivariable logistic regression analysis for good clinical outcome

	Good clinical outcome at three months	
	Adjusted OR (95% CI)	*P*-value
Age	1.005 (0.937–1.078)	*p* = 0.890
Initial NIHSS	0.893 (0.806–0.990)	*p* = 0.031
Procedure time	0.972 (0.947–0.997)	*p* = 0.029
First-line MAT	0.743 (0.124–4.460)	*p* = 0.745
IV tPA	1.225 (0.247–6.089)	*p* = 0.804
Branching type (+)	1.104 (0.145–8.431)	*p* = 0.924
Successful recanalization	1.409 (0.134–14.816)	*p* = 0.775
Atrial fibrillation	0.631 (0.073–5.475)	*p* = 0.676
Hypertension	1.705 (0.303–9.579)	*p* = 0.545

*NIHSS* National Institutes of Health Stroke Scale, *MAT* Manual aspiration thrombectomy, *IV tPA* Intravenous tissue-type plasminogen activator

## Discussion

Acute posterior circulation LVO is an uncommon cause of stroke, but it has been associated with high mortality and morbidity in spite of treatment, when compared with anterior circulation LVO [2, 3]. Recent trials of various mechanical thrombectomy devices used to treat BAO had high recanalization rates (74–100%), with a relatively high rate of good outcomes at 3 months (29–50%) and low mortality rates (12–50%). In our study, the overall successful recanalization rate (78%), good outcome rate (42%), and mortality rate (12%) were similar to those of prior studies [11–16].

One previous study compared clinical and radiological outcomes of patients treated with retrievable stents and the Penumbra 054 suction catheter system [16]. The authors concluded that total procedure time was significantly shorter in the Penumbra suction thrombectomy group (median 101 vs. 53 min). Our study also revealed that the procedure time was reduced by more than half in the MAT group compared to the SR group (median 28 vs. 65 min), and notably these were much shorter procedure times than in the previous study. We believe the main reason for the shorter procedure time in the MAT group is the development of more advanced suction devices. We used a newer suction catheter, such as the Penumbra 5 Max, ACE 68, or Sofia 6F, which have larger inner diameters and a longer distal flexible segment than previous models. Furthermore, delivery of larger-bore catheters to the basilar artery is easier than in the anterior circulation because of the relatively straight vascular course.

Bernava et al. [17] reported that an angle (≥125.5°) of interaction between the aspiration catheter and clot was an important factor for successful clot removal. Consistent with these findings, we observed that the angle between the aspiration catheter and clot was almost 180° due to the straight course of the basilar artery, thus MAT was more effective for basilar artery occlusion than SR, leading to a higher first-pass successful recanalization rate and lower number of passes. Moreover, in technical terms, MAT is a more rapid procedure than SR thrombectomy, which requires repeat attempts to achieve successful recanalization in most cases. Stent re-sheathing and reselection of the occluded artery are necessary if recanalization fails on the first attempt during SR thrombectomy. During MAT, however, if the vacuum was released during catheter withdrawal, we placed a Penumbra reperfusion catheter in the distal VA or proximal BA and performed angiography via that catheter because clots could be sucked into the catheter. If persistent occlusion was observed on angiography, we simply reinserted the microcatheter/microwire into the suction catheter and reselected the occluded artery. We believe this saved a great deal of time.

In our study, the patients treated with SR had a higher frequency of SAH after the procedure (11.7%), with one death due to vessel rupture. There were no cases of postprocedural SAH in the MAT group. These results suggest that blind wire passage can lead to vascular perforation, a possibility that is more dangerous in basilar and perforator vessels of the proximal PCA than in the anterior circulation (Fig. 1b).

Our study showed that only low admission NIHSS and short procedure time, not choice of fist-line treatment method, were associated with favorable clinical outcomes on multivariable analysis. The favorable clinical outcome rates were higher in the MAT group, but the difference did not reach statistical significance. Results of earlier studies on acute vertebrobasilar occlusions identified the initial severity of neurologic deficits, time to treatment, location of the occlusion, degree of collaterals, treatment modalities, and timely reperfusion as key prognostic factors [18–20]. Compared with the anterior circulation, the damage from a BAO is primarily due to injury to the perforator territory, which lacks collaterals and thus may be more susceptible to prolonged time to reperfusion. Newer devices with large-bore catheters may shorten the recanalization time and improve mortality rates as seen in our patients; however, the selection of patients who have a high likelihood of achieving a good outcome is another essential factor. Imaging-based identification of the initial extent of the core may be helpful in selecting patients who will benefit from mechanical thrombectomy [21].

This study has several limitations. First, it was retrospective design, signle center cases, and relatively small number of patients were evaluated. Due to the retrospective nature of this study, the mechanical thrombectomy device was chosen at the discretion of the operator and thus there may be selection bias. A prospective, randomized controlled trial is warranted to demonstrate efficacy of MAT for acute basilar artery occlusion. Second, there was no definite indication or guideline for the choice of first-line mechanical thrombectomy device. The lack of treatment randomization could weaken the results of our comparisons between SR and MAT. Third, we did not consider the effect of pretreatment collateral flow, which can affect outcomes after MT. Further large randomized studies are needed to confirm our findings.

## Conclusion

Selection of MAT using a large-bore aspiration catheter for acute BAO may be a safe and effective first-line treatment method with higher first-pass recanalization rate and shorter procedure time than SR.

## Data Availability

The datasets generated and analysed during the current study are not publicly available, but are available from the corresponding author on reasonable request.

## Abbreviations

SR: Stent retriever
MT: Mechanical thrombectomy
MAT: Manual aspiration thrombectomy
BAO: Basilar artery occlusion
LVO: Large vessel occlusion
AIS: Acute ischemic stroke
SAH: Subarachnoid hemorrhage
NIHSS: National Institute of Health Stroke Scale
mRS: Modified Rankin Scale
CT: Computed tomography
MRI: Magnetic resonance image
mTICI: Modified thrombolysis in cerebral infarction

## References

[1] Leys D (2001). Atherothrombosis: a major health burden. Cerebrovasc Dis.

[2] Phan K, Phan S, Huo YR, Jia F, Mortimer A (2016). Outcomes of endovascular treatment of basilar artery occlusion in the stent retriever era: a systematic review and meta-analysis. J Neurointerv Surg.

[3] Humphries W, Hoit D, Doss VT, Elijovich L, Frei D, Loy D (2015). Distal aspiration with retrievable stent assisted thrombectomy for the treatment of acute ischemic stroke. J Neurointerv Surg.

[4] Turk AS, Frei D, Fiorella D, Mocco J, Baxter B, Siddiqui A (2018). ADAPT FAST study: a direct aspiration first pass technique for acute stroke thrombectomy. J Neurointerv Surg.

[5] Maus V, Behme D, Kabbasch C, Borggrefe J, Tsogkas I, Nikoubashman O (2018). Maximizing first-pass complete reperfusion with SAVE. Clin Neuroradiol.

[6] Maegerlein C, Berndt MT, Monch S, Kreiser K, Boeckh-Behrens T, Lehm M (2020). Further development of combined techniques using stent retrievers, aspiration catheters and BGC : the PROTECT (PLUS) technique. Clin Neuroradiol.

[7] McTaggart RA, Tung EL, Yaghi S, Cutting SM, Hemendinger M, Gale HI (2017). Continuous aspiration prior to intracranial vascular embolectomy (CAPTIVE): a technique which improves outcomes. J Neurointerv Surg.

[8] Lapergue B, Blanc R, Gory B, Labreuche J, Duhamel A, Marnat G (2017). Effect of endovascular contact aspiration vs stent retriever on revascularization in patients with acute ischemic stroke and large vessel occlusion: the ASTER randomized clinical trial. JAMA..

[9] Turk AS, Siddiqui A, Fifi JT, De Leacy RA, Fiorella DJ, Gu E (2019). Aspiration thrombectomy versus stent retriever thrombectomy as first-line approach for large vessel occlusion (COMPASS): a multicentre, randomised, open label, blinded outcome, non-inferiority trial. Lancet.

[10] Zaidat OO, Castonguay AC, Linfante I, Gupta R, Martin CO, Holloway WE (2018). First pass effect: a new measure for stroke Thrombectomy devices. Stroke..

[11] Schonewille WJ, Wijman CA, Michel P, Rueckert CM, Weimar C, Mattle HP (2009). Treatment and outcomes of acute basilar artery occlusion in the basilar artery international cooperation study (BASICS): a prospective registry study. Lancet Neurol.

[12] Espinosa de Rueda M, Parrilla G, Zamarro J, Garcia-Villalba B, Hernandez F, Moreno A (2013). Treatment of acute vertebrobasilar occlusion using thrombectomy with stent retrievers: initial experience with 18 patients. AJNR Am J Neuroradiol.

[13] Mordasini P, Brekenfeld C, Byrne JV, Fischer U, Arnold M, Heldner MR (2013). Technical feasibility and application of mechanical thrombectomy with the solitaire FR revascularization device in acute basilar artery occlusion. AJNR Am J Neuroradiol.

[14] Du S, Mao G, Li D, Qiu M, Nie Q, Zhu H (2016). Mechanical thrombectomy with the solitaire AB stent for treatment of acute basilar artery occlusion: a single-center experience. J Clin Neurosci.

[15] Baek JM, Yoon W, Kim SK, Jung MY, Park MS, Kim JT (2014). Acute basilar artery occlusion: outcome of mechanical thrombectomy with solitaire stent within 8 hours of stroke onset. AJNR Am J Neuroradiol.

[16] Son S, Choi DS, Oh MK, Hong J, Kim SK, Kang H (2016). Comparison of solitaire thrombectomy and penumbra suction thrombectomy in patients with acute ischemic stroke caused by basilar artery occlusion. J Neurointerv Surg.

[17] Bernava G, Rosi A, Boto J, Brina O, Kulcsar Z, Czarnetzki C (2020). Direct thromboaspiration efficacy for mechanical thrombectomy is related to the angle of interaction between the aspiration catheter and the clot. J Neurointerv Surg.

[18] Cross DT, Moran CJ, Akins PT, Angtuaco EE, Diringer MN (1997). Relationship between clot location and outcome after basilar artery thrombolysis. AJNR Am J Neuroradiol.

[19] Voetsch B, DeWitt LD, Pessin MS, Caplan LR (2004). Basilar artery occlusive disease in the new England Medical Center posterior circulation registry. Arch Neurol.

[20] Singer OC, Berkefeld J, Nolte CH, Bohner G, Haring HP, Trenkler J (2015). Mechanical recanalization in basilar artery occlusion: the ENDOSTROKE study. Ann Neurol.

[21] Guillaume M, Lapergue B, Gory B, Labreuche J, Consoli A, Mione G (2019). Rapid Successful Reperfusion of Basilar Artery Occlusion Strokes With Pretreatment Diffusion-Weighted Imaging Posterior-Circulation ASPECTS <8 Is Associated With Good Outcome. J Am Heart Assoc.

